# Accurate long-range forecasting of COVID-19 mortality in the USA

**DOI:** 10.1038/s41598-021-91365-2

**Published:** 2021-07-05

**Authors:** Pouria Ramazi, Arezoo Haratian, Maryam Meghdadi, Arash Mari Oriyad, Mark A. Lewis, Zeinab Maleki, Roberto Vega, Hao Wang, David S. Wishart, Russell Greiner

**Affiliations:** 1 Department of Mathematics and Statistics, Brock University, St. Catharines, ON L2S 3A1 Canada; 2 Department of Electrical and Computer Engineering, Isfahan University of Technology, 84156-83111 Isfahan, Iran; 3 Department of Mathematical and Statistical Sciences, University of Alberta, Edmonton, AB T6G 2G1 Canada; 4 Department of Biological Sciences, University of Alberta, Edmonton, AB T6G 2E9 Canada; 5 Department of Computing Science, University of Alberta, Edmonton, AB T6G 2E8 Canada; 6 Alberta Machine Intelligence Institute, Edmonton, AB T5J 3B1 Canada

**Keywords:** Infectious diseases, Computer science

## Abstract

The need for improved models that can accurately predict COVID-19 dynamics is vital to managing the pandemic and its consequences. We use machine learning techniques to design an adaptive learner that, based on epidemiological data available at any given time, produces a model that accurately forecasts the number of reported COVID-19 deaths and cases in the United States, up to 10 weeks into the future with a mean absolute percentage error of 9%. In addition to being the most accurate long-range COVID predictor so far developed, it captures the observed periodicity in daily reported numbers. Its effectiveness is based on three design features: (1) producing different model parameters to predict the number of COVID deaths (and cases) from each time and for a given number of weeks into the future, (2) systematically searching over the available covariates and their historical values to find an effective combination, and (3) training the model using “last-fold partitioning”, where each proposed model is validated on only the last instance of the training dataset, rather than being cross-validated. Assessments against many other published COVID predictors show that this predictor is 19–48% more accurate.

## Introduction

Coronavirus disease (COVID-19) was declared a public health emergency of international concern in January 2020 by the World Health Organization^1^. Since then, more than 76 million confirmed cases and almost 1.7 million deaths due to COVID-19 have been reported worldwide^2^. Some of these cases and deaths might have been prevented if more aggressive public policies, such as travel restrictions and lockdowns, had been implemented sooner. However, lockdowns have the downside of causing severe economic disruptions. For example, the number of global job losses has been ten times greater than those arising during the first months of the 2008 global financial crisis. It is unlikely that employment in many developed countries will return to pre-pandemic levels before 2022^3^. Throughout the pandemic, governments have been forced to find a balance between public safety and public good, constantly adjusting preventive policies to mitigate the disease spread, while trying to preserve the economy. Robust policy development and planning requires pandemic models that can accurately predict the number of COVID-19 cases and deaths far into the future, as such models would allow governmental policy makers to examine the effect of different preventive policies^4^.

While many epidemic models have successfully explained the dynamics of disease outbreaks after the fact, their success in forecasting has, at best, been mixed^4,5^. Indeed, there have been many failures in forecasting for COVID-19. Early mis-predictions of 100,000,000 USA COVID cases within 4 weeks of the beginning of the pandemic, suggesting sizable impacts on hospital and ICU requirements, greatly misinformed policy makers^1^. The Center for Disease Control (CDC) now maintains a national forecasting site, where 60 modeling groups continually update their forecasts for new COVID-19 cases, hospitalization, and deaths in the USA^6,7^. While these forecasts are better than earlier attempts^8,9^, there is still much room for further improvement, especially for the long-range (5+ weeks) forecasts. These longer range forecasts are important, as they provide guidance and reasonable preparation time for preventive policy makers and health managers.

This paper describes a novel approach that improves long-range forecasting substantially over existing COVID-19 prediction models—predicting new COVID-19 cases and deaths in the US over different time periods, more accurately than existing models from the CDC site^10^. Our forecaster also predicts the weekly periodicity reported in the daily reported number of deaths, and it predicts COVID-19 surges up to 2 months ahead. We believe that similarly accurate COVID-19 prediction models could be developed for other countries if the appropriate input data is available.

Instead of designing a single model with fixed parameters to forecast cases and deaths for the whole pandemic duration, we instead created a general learner, called *LaFoPaFo* (short for *LAst FOld PArtitioning FOrecaster*), that uses “last-fold partitioning” to find the best model parameters, the best combination of these features, and the best history-length to produce the forecasting model. Given the target cases or deaths, and forecast horizon ranging from 5 to 10 weeks, LaFoPaFo produces a model (involving a learned subset of existing available epidemiological data) that is designed to forecast this target, at this forecast interval in the future. LaFoPaFo considers 11 different features, including the current number of COVID-19 tests, cases, and deaths, social activity measures, and weather-related covariates specific to the USA. It also includes their historical values at the earlier weeks, back to the start of the pandemic in the US. Here, we compare the forecasting results of our LaFoPaFo with those available at CDC^10^ during the 7 weeks starting from September 27 to November 14, 2020. We also assess the design features of our learning approach. The SI provides the performance data of our model for an extended range (Supplementary Figures 1–6).

## Results

**Figure 1:**
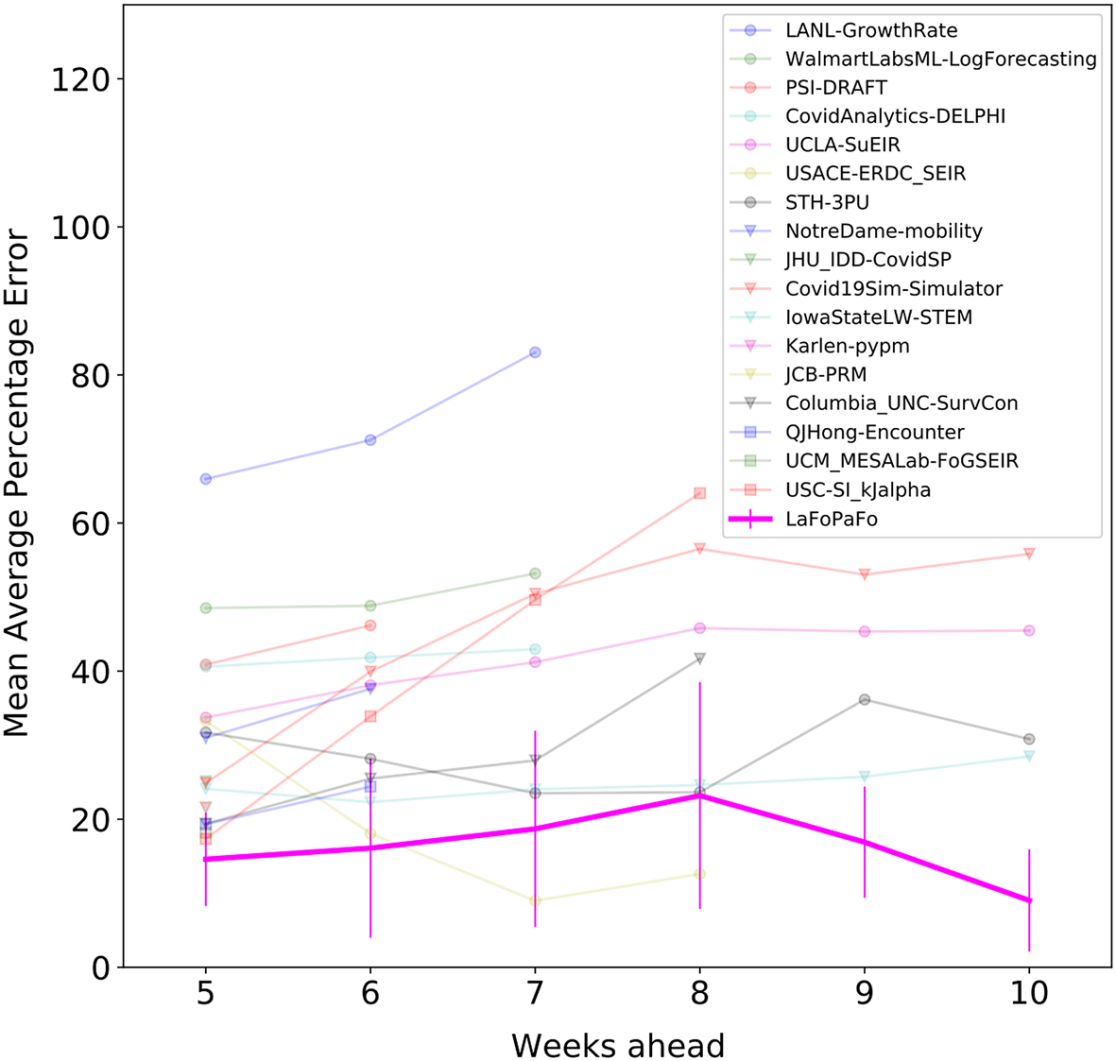
Forecast accuracy of the projected weekly number of reported COVID-19 deaths in the US. The accuracies of the 17 predictive models submitted to the CDC, as well as LaFoPaFo, on forecasting the weekly average (non-cumulative) number of deaths in the US for each of the 7 weeks starting from September 27 to November 14, 2020. We could only consider six test weeks for the future 10-weeks predictions as insufficient training instances were available before the week September 21–27. For each of these 7 weeks, we recorded the forecasts made by each model, for 5 to 10 weeks ahead, and compared each forecast to the true value. We then computed the MAPE for each model and time-horizon as the average over the 7 weeks, depicted with a single point in the graph. The vertical purple lines are the error bars of LaFoPaFo and are computed as the standard deviation over the seven MAPEs. Note the forecasting results for the CDC models were taken from the tables at the forecast hub website. Most CDC models did not submit forecasts for long-range horizons; only five models submitted the forecast results for the future 10 weeks. We see that LaFoPaFo is the most accurate forecaster over the 5, 6, 9 and 10 week forecast horizons, with a significant difference for 9 and 10 weeks.

We first consider forecasting the weekly, averaged number of COVID-19 deaths. Figure 1 shows that our LaFoPaFo outperforms all CDC models for essentially every forecast horizon from five to 10 weeks. The only exception is the USACE-ERDC_SEIR model^11^, which outperforms LaFoPaFo for future 7 and 8 weeks. In particular, for predictions extending to 10 weeks, LaFoPaFo's mean absolute percentage error (MAPE) is 9%, while the best second model has an error of 28%. Very few research groups provided their forecasting results for more than 8 weeks ahead; those that did, report an accuracy that decreased dramatically as the forecasting length increased.

**Figure 2:**
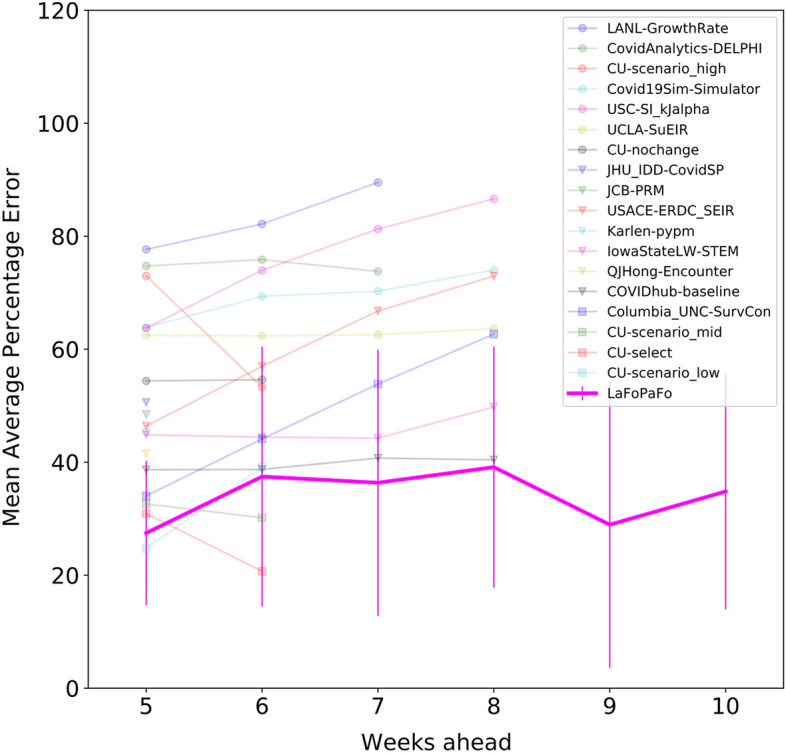
Forecast accuracy of the projected weekly number of reported COVID-19 cases in the US. The settings are the same as Fig. 1, but here the models are evaluated on forecasting the future number of reported COVID-19 cases. LaFoPaFo outperforms other models over almost every forecasting horizon. None of the CDC models provided forecasting results for future 9 and 10 week horizons.

Figure 2 shows a similar trend for predicting the number of reported cases, where LaFoPaFo is in the top three at all times, and the best, often by a large margin, starting with a 7-week forecast. The CDC models that were best at forecasting the number of deaths were often less accurate at forecasting the number of confirmed cases. This was also true for LaFoPaFo, but not as significantly. The MAPE of the USC-SI_kJalpha, which was the second-best predictor for 5-week reported cases, increased from 17% (deaths) to 63% (cases); LaFoPaFo, on the other hand, increased from 14% to just 27%. Although seemingly large, the error bars are based on only seven values. Moreover, the error bars of LaFoPaFo are in the same range of values as most other CDC forecasters.

As a more in-depth comparison, Table 1 presents the “p < 0.05 2-sided t-test” results with the null hypothesis that the MAPEs of LaFoPaFo and each CDC model were drawn from the same distribution. For predicting the number of deaths, LaFoPaFo won 22 times (rejected the null hypothesis), lost 0 times, and was tied with the CDC models 26 times (i.e., failed to reject the null hypothesis). For predicting the number of cases, LaFoPaFo won 30 times, lost 0 times, and was tied with the CDC models 25 times.

**Table 1:** T-test results.

	#deaths			#cases		
	Win	Tie	Lose	Win	Tie	Lose
5 weeks	8	9	0	10	8	0
6 weeks	8	5	0	4	10	0
7 weeks	6	4	0	5	4	0
8 weeks	3	4	0	3	4	0
9 weeks	2	2	0	–	–	–
10 weeks	3	1	0	–	–	–
Total	30	25	0	22	26	0

The null hypothesis was that the MAPEs of LaFoPaFo and the CDC models follow the same distribution. The test was performed for each of the future 5, 6, …, 10 future weeks forecasts, comparing LaFoPaFo to each of the CDC models. “Win” means that the average MAPE of LaFoPaFo is lower than the CDC model and that the difference is statistically significant. “Tie” means that the difference in MAPE is not statistically significant. “Lose” means that the MAPE of the CDC model is lower and that the difference is statistically significant. LaFoPaFo never lost against any of the CDC models.

**Figure 3:**
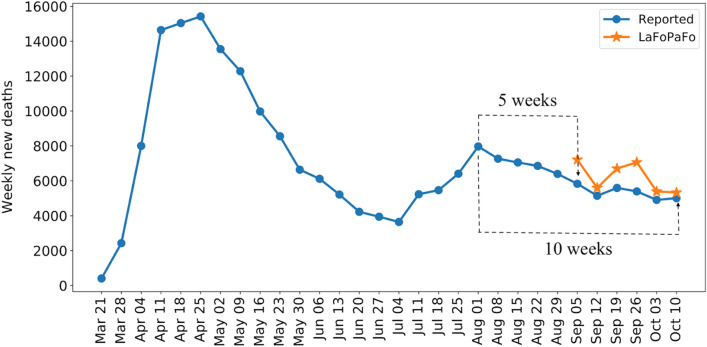
LaFoPaFo's projected number of deaths from 5 to 10 weeks in the future. The forecasts are made based on data accumulated to the week of August 1, 2020.

**Figure 4:**
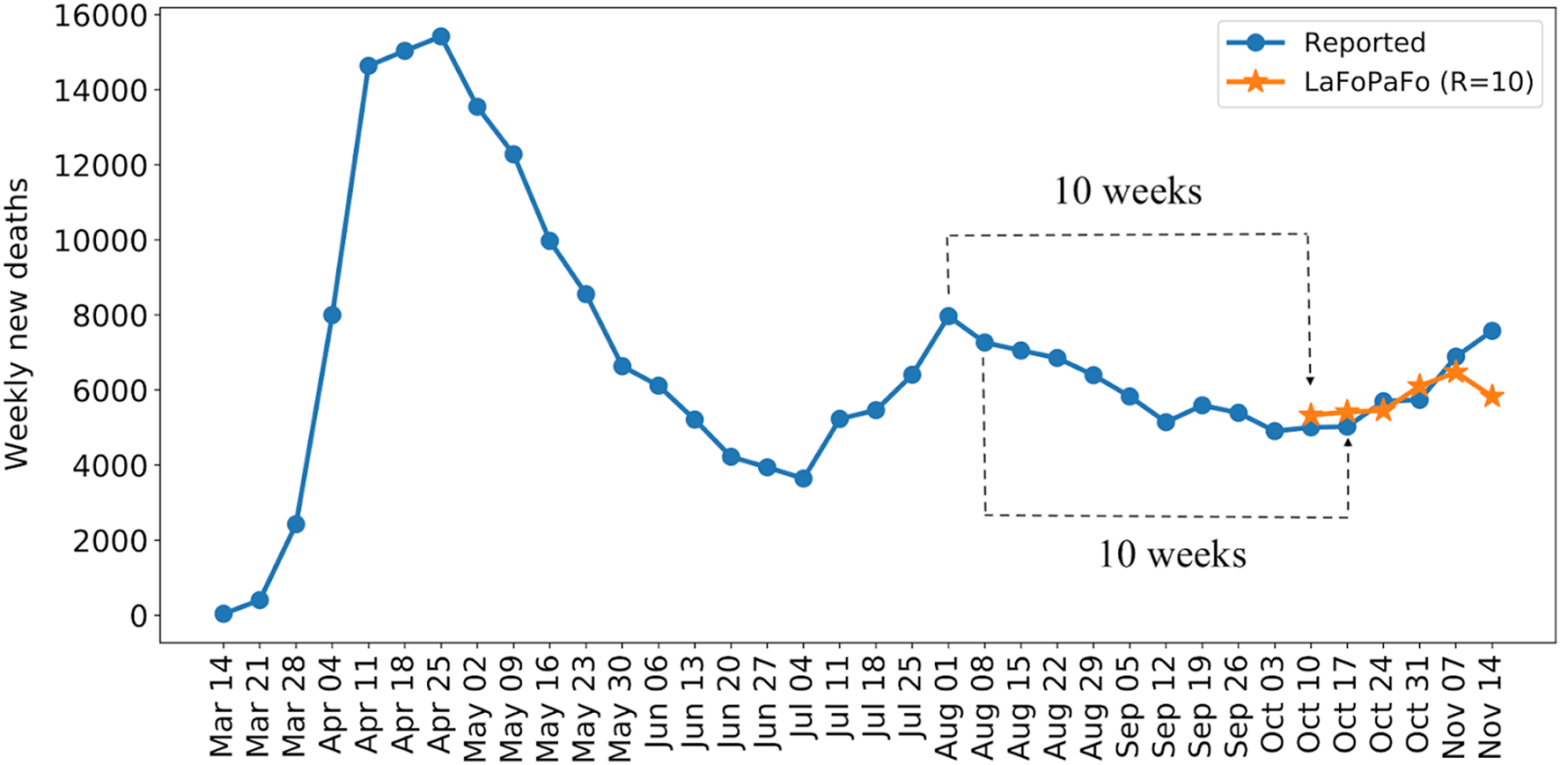
LaFoPaFo’s weekly forecasting results for the number of deaths, ten weeks later, in the US. Note the figure shows only two of the six “10 weeks” lines.

Figure 3 presents the future 5- to 10-week forecasted number of COVID-19 deaths, when LaFoPaFo uses data up to the week of July 26–August 1, 2020. As seen here, LaFoPaFo was able to forecast the almost-exact values of the number of COVID weekly deaths, over the next 6, 9 and 10 weeks. Also, as seen in Supplementary Figure 1, on June 21–27, when the first COVID death wave was damping, LaFoPaFo almost exactly forecasted the peak of the second COVID wave that happened 5 weeks later on July 26–August 1. Moreover, on July 26–August 1, it forecasted that the total number of deaths will decrease by 3000 individuals 10 weeks later (Fig. 4).

**Figure 5:**
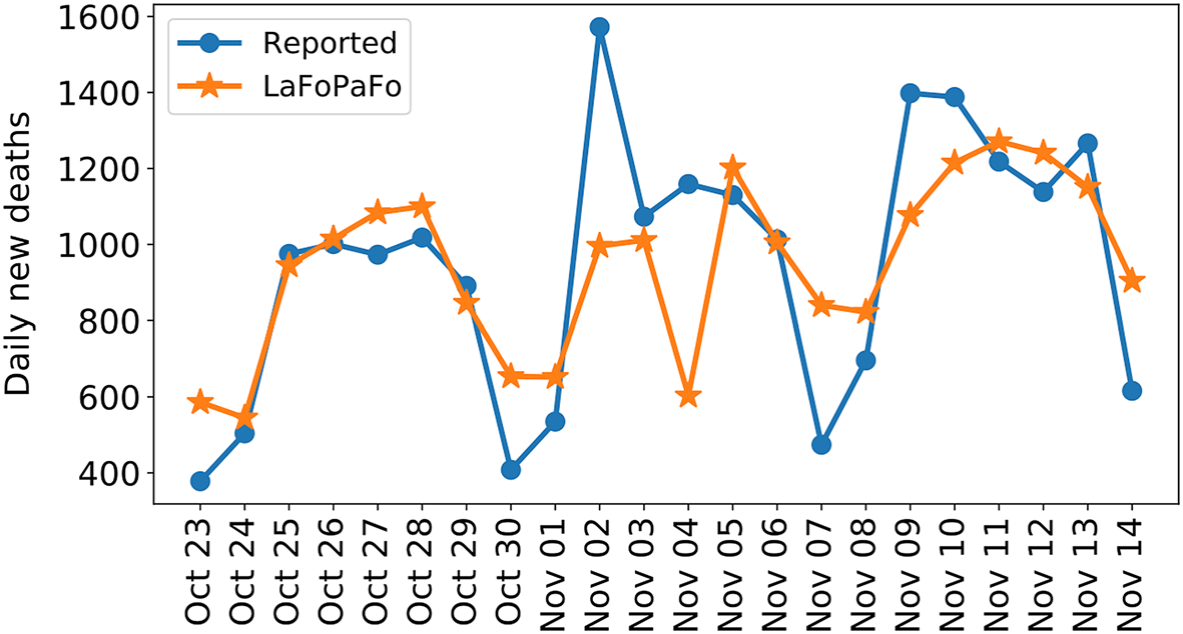
LaFoPaFo’s daily forecasting results for the future 14-day number of deaths in the USA. LaFoPaFo forecasted each day from September 20 to October 10, 2020, 2 weeks ahead. LaFoPaFo forecasts the periodicity in the daily data as well as the peak values with at most one day delay.

Figure 5 presents the future 14-day forecasting results for the daily number of deaths over the 21 days of October 23 to November 14, 2020. This illustrates how LaFoPaFo correctly forecasts the periodicity in the daily data, 2 weeks ahead.

**Figure 6:**
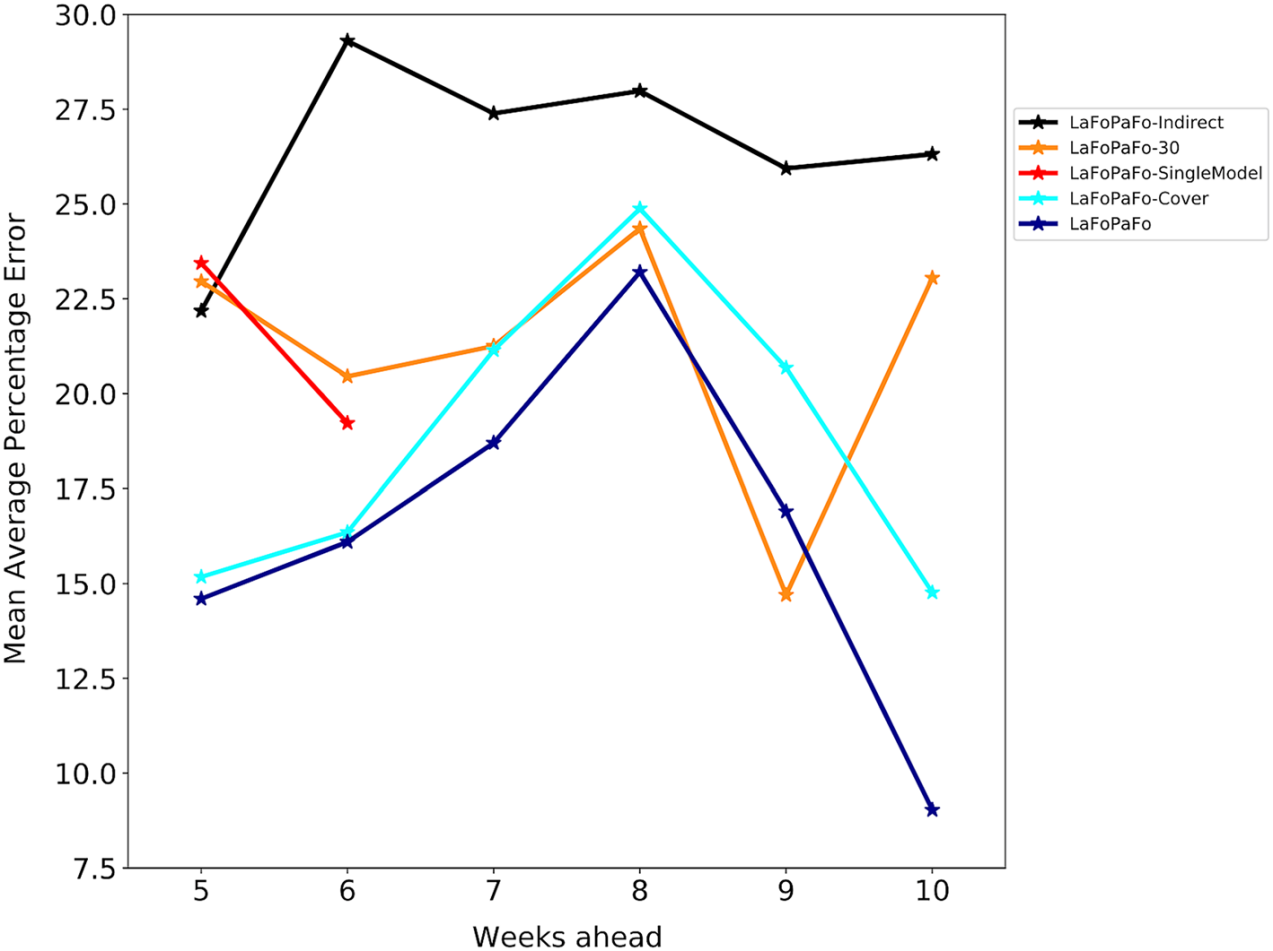
Ablation system, showing the LaFoPaFo's Forecast Accuracy without various design factors. Evaluating the performance on forecasting the future number of COVID-19 deaths from 5 to 10 weeks in the future for LaFoPaFo and its modified versions (that each modify one of its design features), using the same setup as in Fig. 1. The red graph (LaPoFaPo-SingleModel) corresponds to the case where, instead of producing seven forecasting models for the seven test weeks, a single model was produced to forecast all of the 7 weeks. A validation dataset of seven instances (weeks) was used, which limited the forecast horizon to 6 weeks due to the limited training data. The cyan graph (LaPoFaPo-Cover) corresponds to the model where instead of using 11 features, only the previous number of deaths is used. The orange graph (LaPoFaPo-30) corresponds to the model where rather than the last instance, 30% of the training dataset was used as the validation set to choose the final model. The black graph (LaPoFaPo-Indirect) corresponds to the system that forecasts each week in the future based on the intermediate week forecasts, rather than being directly forecasted.

To better understand why LaFoPaFo worked so well, we performed a simple ablation study, where we modified each of its design features, one-by-one (Fig. 6). We found that LaFoPaFo's performance decreased at almost every forecast horizon if any of its design features were altered. Among the alternate models we studied, *(1) LaFoPaFo-SingleModel* that had to produce a single nearest neighbor to forecast all seven test weeks; this version had 23% MAPE for future 5-week forecast rather than the original 15% MAPE. *(2) LaFoPaFo-Cover* that only used the previous number of deaths but no other covariates; its performance would degrade from 17 to 21% MAPE for future 9-week predictions. *(3) LaFoPaFo-30* that used a single 30% validation fold, rather than the one point; this version had 23% MAPE rather than 15% for the future 5-week predictions. *(4) LaFoPaFo-Indirect* that forecasted week one, based on which it forecasts week two, and so on until it forecasts the week of interest based on its own forecast of the previous week; its performance was worse than LaFoPaFo's by at least 10.5% MAPE over the 6 multi-week predictions.

## Discussion

The COVID-19 pandemic has caused dramatic increases in deaths, dire economic fallout and enormous social problems. Many of these consequences were not predicted, but many could have been mitigated if the number of future COVID-19 cases and deaths could have been forecasted accurately. Long-term (2–3 months) pandemic and epidemic forecasts are more challenging than short-term (1–2 weeks) ones, but they are also more essential, as such long-term forecasts are key to effectively planning, developing, implementing and assessing pandemic response policies by governments and public health agencies. However, while many existing COVID-19 prediction models can make 1- or 2-week forecasts reasonably well, most fail at longer forecast horizons. Our machine learning approach can provide long-range forecasts of COVID-19 cases and deaths, up to 10 weeks in the future.

Most mechanistic models, such as the SEIR (susceptible, exposed, infectious, removed) model, use fixed parameters to implicitly model the effect of stochastic, yet influential variables. For example, few models include weather or average number of encounters as stand-alone variables, but many can incorporate them into the infection ratio parameter. Under the assumption that those variables remain constant over time, modelers can project the future number of COVID deaths and cases. This assumption appears approximately valid for the near future, say 1 week, but not for 4 weeks or more. This may explain why some of the submitted CDC models that accurately forecast short-range mortalities, did not submit forecast results for 5-week and longer forecast horizons.

LaFoPaFo's strong forecasting power allows it to effectively predict future COVID-19 surges many weeks ahead. For example, on the week June 21–27, when the first US COVID surge was damping and still had one more week to reach its minimum, LaFoPaFo still predicted that 5 weeks into the future, there would be a second wave. Our model could also forecasted from August 1, when the number of COVID deaths in the US had peaked, that 10 weeks later the number of deaths will decrease by about 3000 individuals over the week. Such long-range predictions can greatly inform public health management.

The fact that LaFoPaFo's base learner uses one of the simplest possible models, namely “*k* nearest neighbor”, emphasizes the strengths of its design features. To forecast the number of cases in the future, a *k* nearest neighbor simply finds the *k* instances in the training dataset that have the most similar covariates, and then returns, as its forecast, the average of their number of cases. This *k* nearest neighbor model is clearly much simpler than those SEIR models with their complicated coupled differential equations that attempt to capture the spread dynamics in the near future, However, LaFoPaFo's approach still enables it to outperform SEIR models in the long-range predictions.

The key to LaFoPaFo's success is its internal training process, which makes critical decisions about the model based on the situation to which that model will be applied, when it is making its forecasts. Here we summarize the five most novel and important aspects of LaFoPaFo.

First, unlike the common approach of producing a single model with fixed parameters to forecast the entire pandemic, the LaFoPaFo has the option of producing different models for forecasting each time interval during the pandemic—that is, “5-week case forecaster” for May 1 may be different from the “5-week case forecaster” for September 20. This makes particular sense for machine-learning approaches where the pandemic dynamics can vary significantly over time—which is true for this COVID-19 pandemic. Our empirical study confirmed that the accuracy of our COVID-19 predictions drops considerably if we require that LaFoPaFo uses only a single model over all times.

Second, like most machine learning tools, LaFoPaFo produces a model that can use many covariates to produce its forecast. LaFoPaFo actually considers eleven different covariates, at multiple time points. If the historical data has five time points, there are fifty-five variables to consider. Then, a feature selection algorithm would have to implicitly consider all 2^55^ combinations. To reduce the chance of overfitting and to save computational time, LaFoPaFo instead considers a systematically-constructed fixed subset of covariates, where all of the historical values of the selected covariates are included, counting from the current times^12^.

Third, to decide on the appropriate hyper-parameters, such as the ‘*k*’ for *k*-nearest-neighbors and the set of features to include, machine learning algorithms often partition the training dataset into sub-training and validation subsets. They then train various models (each with some setting of those hyperparameters) on the sub-training subset, and evaluate the quality of this setting on the validation subsets^13^. This partitioning is typically done randomly, which here means the validation subset will be randomly selected from the weeks within the training subset. The validation subset might, hence, include data that occurs before some data in the sub-training set. This approach is implicitly based on the assumption that the data is iid (independent and identically distributed), implying the distribution of features and outcomes is the same for June 10 as it is for September 30. However, we know that this is not the case, due to differences in policies, “Covid fatigue” and other time-varying unknown influential factors. This is why LaPoFaPo instead uses “last-fold partitioning”, where only the last week of the dataset is taken as the validation and the rest serves as the training dataset. This is also why LaFoPaFo does not use internal cross validation. The idea behind this is to mimic the situation that the learned forecasting model will face in making real predictions, as that model needs to make its predictions based only on data available at that time, and cannot base its model on future data, which is unavailable.

Fourth, for the same reason as described above, we set the size of the validation dataset to be a single week, as the learned system will be asked to focus only on a single value. Increasing the size of the validation means the learned model will prefer hyper-parameters that fit some of the former weeks as well as the “final” week. Given how quickly this pandemic changes, as we only care about the latest date, the validation subset should only be the single “closest” date.

Fifth, we did not follow the common approach of predicting a point in the future by first predicting the intermediate points one by one and using the prediction results of the previous point in predicting the next point. For example, in order to forecast week *t* + 10, that approach would first use previous data (pre-week *t*) to forecast week *t* + 1, then use that prediction result at week *t* + 1 (as well as pre-week *t*) to predict week *t* + 2, and so on. Given that our prediction for week *t* + 1 is just our prediction (and not true information), this can easily accumulate the error. Instead, we trained the “*r*-week horizon” LaFoPaFo with the dataset consisting of the covariates at time t but set the target variable to be the quantity at time *t* + *r*. The better performance of this direct approach is partly explained by comparing the joint probability distribution of the mortality count at two consecutive weeks and the probability distributions of each of the 2 weeks. The maximum of the joint probability does not necessarily equal the joint maximums of each of the probability distributions. Therefore, if the goal is the maximum of the probability distribution of the second week (*r* = 2), that of the first week (*r* = 1) should not be considered.

We close this section with three comments. First, there is no “leakage” from test data to training data. That is, even though LaFoPaFo (for each of the cases, and deaths) is learning a different model for each time and for each forecast time horizon, the learning process uses ONLY the data available at the forecasting start date. Although seemingly trivial, a huge body of literature ignores this when presenting or assessing their results.

Second, while this paper discusses only the task of forecasting weekly COVID-19 cases and deaths for the USA, LaFoPaFo should be applicable to making daily COVID-19 forecasts for other countries, as well as for smaller sub-regions such as counties and states. To facilitate this, we have made the code for the LaFoPaFo approach publicly available.

Third, in addition to allowing health managers and policy makers to plan COVID-19 mitigation, the approach used in this paper opens the door to future research on examining the long-range effects of different preventive policies, allowing policy makers to experiment and plan COVID-19 mitigation decisions.

## Methods

Our goal is to accurately forecast the weekly number of reported COVID-19 cases and deaths over a range of time horizons, varying from 5 to 10 weeks. Our focus is on the United States. Given each specific forecast time interval, we want a model that can use the existing covariates, measured up to the “current” time, to forecast the weekly number of reported COVID-19 deaths or cases at a specific number of weeks in the future. Rather than producing a single predictive model for the entire pandemic duration, we have designed a learner, LaFoPaFo, that learns from data and produces a nearest neighbor model suited to the time period of interest; it then uses that time-duration-target model for this single prediction. For example, the model for “forecasting COVID-19 cases 6-weeks out, from Apr. 1” is different from the model for “forecasting COVID-19 cases 6-weeks out, from Sept. 1”.

### Data

We use a dataset containing 11 features: (1) the number of reported COVID-19 cases and (2) deaths in the US for each day, from the beginning of the disease outbreak in the US (January 22, 2020) until November 14, 2020. This dataset also includes the following covariates: (3) country average number of daily COVID-19 tests, calculated as the number of daily performed tests, averaged over all US states, (4) country average daily temperature and (5) daily precipitation, calculated by averaging daily temperature and daily precipitation over all US counties, as well as several social distancing related covariates. To estimate the relevant social distancing covariates, we used Google mobility data, which contains cell-phone-derived information on the mobility trends of individuals visiting different places including (6) parks, (7) transit stations, (8) residences, (9) workplaces, (10) grocery stores and pharmacies, (11) retail shops and recreation centers. This data is provided from March 1 to November 17. Each of these features shows a change in the number of daily visitors to these places compared to the baseline, where the baseline value for each date was determined based on the day of the week and is equal to the median number of visitors on that day of the week in the 5-week period from January 3 to February 6, 2020. Since the start date of Google Mobility data is from March 1, we only include the dates from March 1 to November 17.

### The learner

In order to forecast the number of reported COVID-19 deaths and cases, LaFoPaFo uses the eleven available covariates in the above dataset and explores their historical values to produce a predictive model. In particular, we focus on forecasting the 7 weeks starting from the week September 27–October 3 to the week November 8–14, which we call our testing dataset. Note this contains seven points. For each of these target times, for each forecast horizon (of r weeks, for *r* ∈ {5,6,7,8,9,10}), and each target variable (case or deaths), LaFoPaFo learns a *k*-nearest neighbor (KNN) model that is trained on the data corresponding to the previous weeks up until r weeks earlier, and sets its last week as the validation dataset and the remaining former weeks as the training dataset. For example, to produce the 6-week COVID-19 death forecaster for October 18–24, LaPoFaPo would have access to the data before September 12. Specifically it would use September 6–12 as the validation set, and train on data before September 5.

LaFoPaFo trains the KNN on the “sub-training data”, with a subset of the covariates, and their historical values, as well as different “*k*” (number of neighbors), resulting in a number of different models. The performance of the models are then evaluated on the single validation point, and LaPoFaPo selects the model that has the best MAPE here. Then that single winning model with the selected parameters is trained on the whole training set (both the sub-training and validation datasets) to produce a final model, which is then used to forecast the single point in the testing dataset.

More specifically, for week *t*, let *Y*(*t*) be the target variable, which is either the number of reported COVID-19 deaths or cases and *X*(*t*) = {*x*_1_(*t*), …, *x*_*C*_(*t*)} be the set of available covariates for time *t* (here *C* = 11; see the Data section above). Each forecast instance involves forecasting, from time *t*, the target variable at a time *r* weeks in the future *Y*(*t* + *r*), where *r* ∈ {5,6,…,10}, from the dataset containing the target variable and available covariates from the beginning week in our dataset to week *t*, *D*(*t*) = {*Y*(*t*ʹ + *r*), *X*(*t*ʹ)}_*t*ʹ=1_^*t*-*r*^. We refer to each historical value of a covariate as a feature. So a covariate is some *x*_i_(*t*) but a feature can be *x*_i_(*t*-*h* + 1) for any history *h* = 1,2,…. The learner executes the following steps for each week *T* of the testing dataset, to be forecasted *r* weeks ahead.

**Figure 7:**
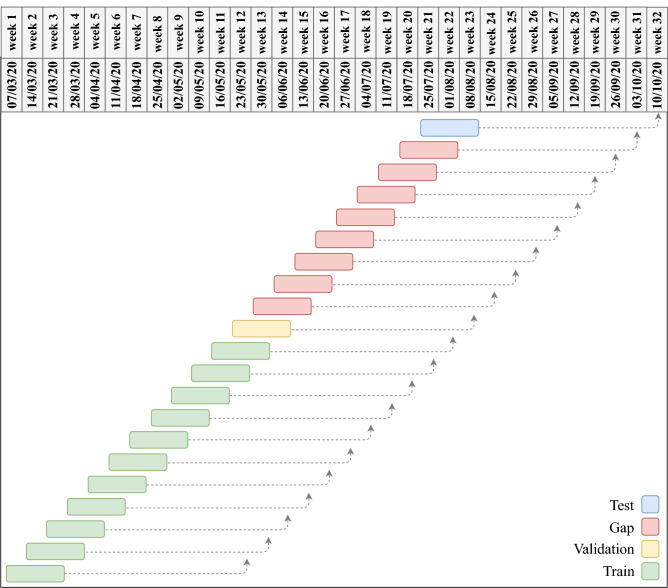
Partitioning the dataset into train, validation and test sets. The forecasting task is to use the covariates at weeks *t*, *t*-1, *t*-2 to forecast the target variable at week *t* + 9. So the forecast horizon is nine (*r* = 9) and the history length is three (*h* = 3). Each row in the graph corresponds to an instance. The length of the dashed arrows are the forecast horizon and the length of the rectangles are the history length. The last instance is taken as the test dataset; that is, to forecast week October 3–10 using the covariates on weeks July 25 to August 8. The earlier eight red instances are not used and the yellow one before is used as the validation data instance. The green instances form the training dataset.
Step 2: Rank the covariates according to the *minimum redundancy maximum relevance (mRMR)* method, based on the value of the target variable and over the whole training plus validation data on the covariates. This results in some ordering *x*_1_(*t*) >  *x*_2_(*t*) > … > *x*_*C*_(*t*) of the covariates with respect to *Y*(*t* + *r*), renumbering as necessary, for *t* from 1 to *T*-*r*.
Step 3: Constructs the following covariate sets: Set#1 containing only the top ranked covariate; Set#2 containing both the first and second top ranked covariates, and so on until the last set containing all of the covariates. This results in the covariate sets
  $$ \left\{ {{ {x}}_1\left( { {t}} \right)} \right\}, \, \left\{ {{ {x}}_1\left( { {t}} \right),{ { x}}_2\left( { {t}} \right)} \right\}, \, \ldots , \, \, \left\{ {{ {x}}_1\left( { {t}} \right),{ {x}}_2\left( { {t}} \right), \ldots ,{ {x}}_C\left( { {t}} \right)} \right\}. $$
  LaFoPaFo considers just these *C* different possible covariate sets. Note, for example, the combination of the first and third covariates {*x*_ 1_(*t*), *x* _3_(*t*)} is not allowed (Supplementary Table 1).
Step 4: Extend each of the above covariate sets by including their values at one, two, … up to five, former weeks. We call each of the resulting sets, a feature set*.* That is, LaFoPaFo considers the following 5×C feature sets:
  $$ {\text{history-length }} = { 1}:\left\{ {{ {x}}_1\left( { {t}} \right)} \right\},\left\{ {{ {x}}_1\left( { {t}} \right),{ { x}}_2\left( { {t}} \right)} \right\}, \ldots , \, \left\{ {{ {x}}_1\left( { {t}} \right), { {x_2}}\left( { {t}} \right)\ldots ,{ {x}}_C\left( { {t}} \right)} \right\}, $$
  $$ {\text{history-length }} = { 2}:\left\{ {{ {x}}_1\left( { {t}} \right),{ {x}}_1\left( {{ {t}} - {1}} \right)} \right\},\left\{ {{ {x}}_1\left( { {t}} \right),{ {x}}_1\left( {{ {t}} - {1}} \right),{ { x}}_2\left( { {t}} \right),{ { x}}_2\left( {{ {t}} - {1}} \right)} \right\}, \ldots ,\left\{ {{ {x}}_1\left( { {t}} \right),{ {x}}_1\left( {{ {t}} - {1}} \right), \ldots ,{ {x}}_C\left( { {t}} \right),{ {x}}_C\left( {{ {t}} - {1}} \right)} \right\}, \ldots $$
  $$ {\text{history-length}} = 5:\left\{ {{ {x}}_1\left( { {t}} \right), \ldots ,{ {x}}_1\left( {{ {t}} - {4}} \right)} \right\}, \, \left\{ {{ {x}}_1\left( { {t}} \right), \ldots ,{ {x}}_1\left( {{ {t}} - {4}} \right),{ { x}}_2\left( { {t}} \right), \ldots ,{ {x}}_2\left( {{ {t}} - {4}} \right)} \right\}, \ldots,\left\{ {{ {x}}_1\left( { {t}} \right), \ldots,{ {x}}_1\left( {{ {t}} - {4}} \right), \ldots ,{ {x}}_C\left( { {t}} \right), \ldots ,{ {x}}_C\left( {{ {t}} - {4}} \right)} \right\} $$
  The input of the final learned model will be the variables of one of these feature sets. Hence, LaFoPaFo is seeking the optimal number of covariates and history-length. (Supplementary Table 1 shows LaFoPaFo used h* = 3 and c* = 7).
Step 5: Construct the sub-training, validation, and test datasets corresponding to each feature set. Each of these datasets consist of one column for the target variable and *ch* other columns for the features, where *c* is the number of covariates selected, and *h* is the history-length used in that feature set. The rows of each of these datasets correspond to the same rows as in the raw datasets.
Step 6: For each number of covariates *c* and history-length *h*, train a *k* nearest neighbor on the sub-training dataset*.* Using the scikit-learn package, the value of *k* is chosen from the range of 10 to 200 by applying a five-fold cross-validation on the sub-training dataset. Note that the cross-validation is only on the sub-training, not the validation, nor the testing datasets.
Step 7: Evaluate each of the trained models on the validation dataset to find the best-at-validation model—ie, the parameters *k*, *c*, *h* that had the lowest absolute error.
Step 8: Re-train the best-at-validation model on the whole sub-training and validation dataset with the same optimal, *k*-value, number of covariates and history length that were chosen in Step 7. This is the final model that the learner produces to forecast the target variable *Y*(*T* + *r*) at the single instance of the testing dataset, with the given forecast interval *r*.

Step 1: Construct the “raw” test instance, consisting of the target variable at the test week *T* and the covariates *r* weeks earlier; that is {*Y*(*T*), *X*(*T*-*r*)}. We call this instance “raw” as it does not include historical values of the covariates. Out of the remaining earlier instances, eliminate the most recent *r*-1 to obtain the raw training dataset; that is *D*(*T*-*r*). Take the last instance of the training dataset, i.e., *last-fold partition*, as the raw validation set, and the remaining as the raw sub-training. So the raw validation will be the instance {*Y*(*T*-*r*), *X*(*T*-2*r*)} and the raw subtraining will be *D*(*T*-*r*-1). Namely, the data at week *T*-*r* will be the raw validation and the data at weeks 1, …, *T*-*r*-2, *T*-*r*-1will be the raw training dataset. See Fig. 7 for an example.

We repeat this procedure on each of the seven points in the testing dataset and take the mean to obtain the mean absolute percentage error (MAPE) on the testing dataset. In line with other performance measures^14^, for each forecast horizon, we calculated the standard deviation of the MAPE of the 7 predictions. This resulted in the error bars in Figs. 1 and 2.

### Comparison to existing models

The Center for Disease Control and Prevention (CDC) has provided a platform that allows various research teams to submit their forecasts of the weekly number of COVID-19 deaths and confirmed COVID-19 cases in the US. We compared our final model with those COVID-19 prediction models provided at the CDC website, for each target (cases or deaths), and for each forecast horizon (5 to 10 weeks). For each range *r* and target, we included only those CDC models that have provided forecasts at least six out of the seven testing instances. Note that some models only predicted for a subset of the *r* values. For those with forecasts for just 6 weeks, we calculated the MAPE over the provided 6 weeks. The forecasting performance statistics, including the start and end dates, and definition of the epidemiological week, was consistent with our setup for LaFoPaFo.

To make a more in-depth comparison, we conducted the following set of 2-sided *t*-tests. For each outcome (cases, and deaths), each CDC model, and each forecast horizon *r*, we took two samples: (1) a vector of size seven, consisting of the MAPE values of LaFoPaFo over each of the seven test weeks, and (2) a vector of size seven, consisting of the MAPE values of that specific CDC model over each of the seven test weeks. We then ran the *t*-test with the null hypothesis that both samples arise from the same distribution.

### Assessing the design of the learner

To assess the different design features of LaFoPaFo, we conducted a simple ablation test, where we modified each design feature at a time. To assess whether a feature produces a possibly different model for each of the seven test points, we compared the performance to when the learner produced only a single model, that it applied to all seven test points. To follow the last-fold partitioning, we increased the “validation fold” to 7 weeks so that it equals the size of the “testing fold”. To assess the effect of having a single fold as the validation, we re-ran LaFoPaFo when the validation size was increased to 30% of the dataset after removing the 7 weeks corresponding to the held-out test set. To assess the effect of forecasting the week of interest directly, we compared the results to when it predicted step by step, where it first predicts week one, and then uses the resulting prediction to predict week 2, and so on. Finally, to assess the contribution of the covariates to the prediction of our final model, we applied our learner to the reduced dataset consisting of only the death covariates and called the resulting model, the one-feature model.

### Daily forecasting

Above, we asked LaFoPaFo to forecast the weekly average number of cases. We also assessed the performance of LaFoPaFo at making daily COVID-19 case and death predictions. We followed the same setup used for the weekly predictions, but instead used daily data. We used the 21 days from September 20 to October 10, 2020 as the test points, with a maximum history length of 10 days and a forecast horizon of 14 days.

### Use of experimental animals, and human participants

The data used in this study is collected from publicly available resources, and the authors did not perform any human or animal experiments.

## Supplementary Information

Supplementary Information.

## Data Availability

The number of deaths and confirmed cases are taken from Johns Hopkins University COVID-19 data repository^15^. The google mobility data is taken from google reports of community mobility^16^. The temperature and precipitation is taken from Daily Summaries dataset^17^. A more detailed version of the dataset is under review^18^ and is available online^19^.

## Code availability

The codes are available at our Git repository https://github.com/network-and-Data-Science-IUT/covid-19^20^.
