# Supplementary material for: Accurate long-range forecasting of COVID-19 mortality in the USA
Source: Sci Rep. 2021 Jul 5;11:13822. doi: 10.1038/s41598-021-91365-2 (PMC8257700; doi:10.1038/s41598-021-91365-2)
Supplement: Supplementary file 1 — Supplementary Information. [file 41598_2021_91365_MOESM1_ESM.pdf]

Accurate long-range forecasting of COVID-19 mortality in the USA

Pouria Ramazi, Arezoo Haratian, Maryam Meghdadi, Arash Mari Oriyad, Mark A. Lewis, Zeinab Maleki, Roberto Vega, Hao Wang, David S. Wishart, Russell Greiner

Supplementary information

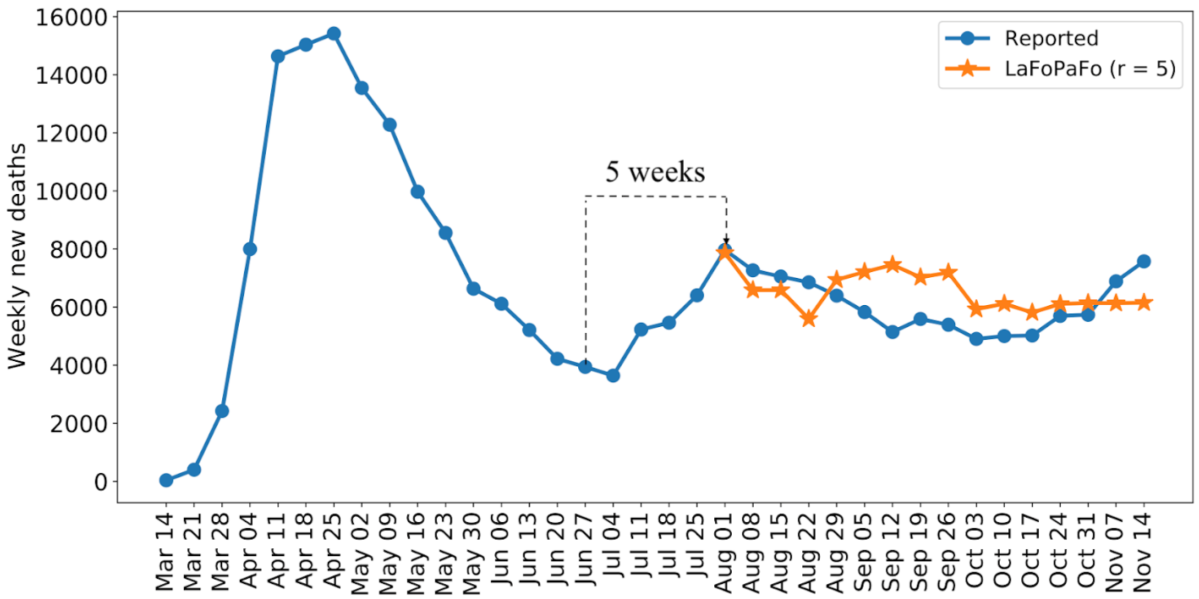

Supplementary Figure 1. LaFoPaFo's future five-week forecasts of the weekly number of deaths in the US. Each orange point in the graph was predicted five weeks earlier.

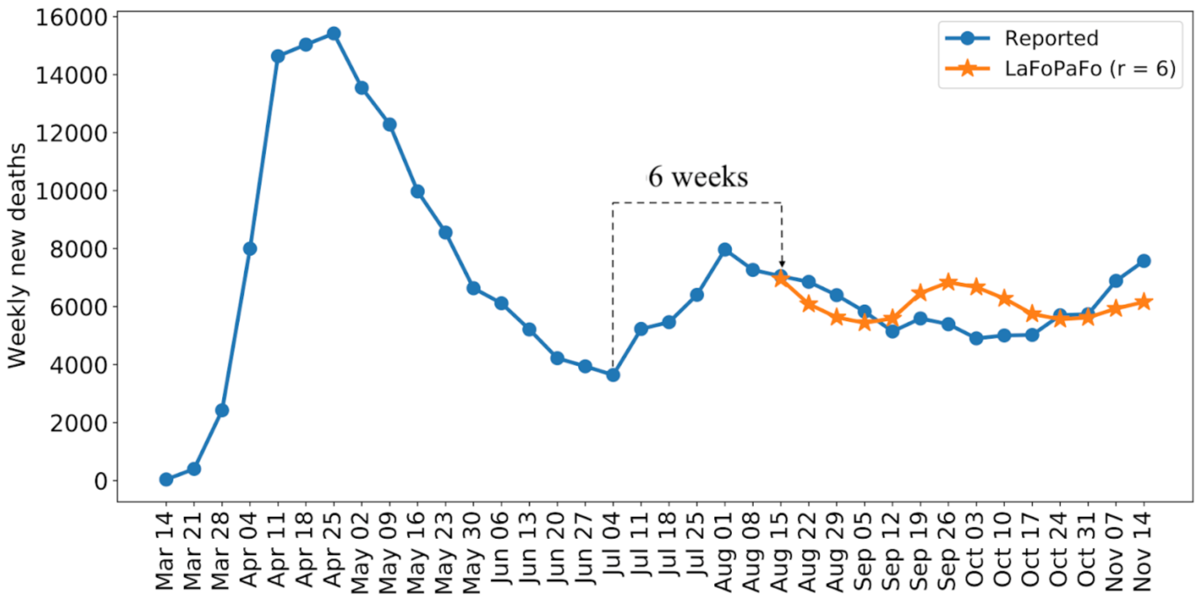

Supplementary Figure 2. LaFoPaFo's future six-week forecasts of the weekly number of deaths in the US. Each orange point in the graph was predicted six weeks earlier.

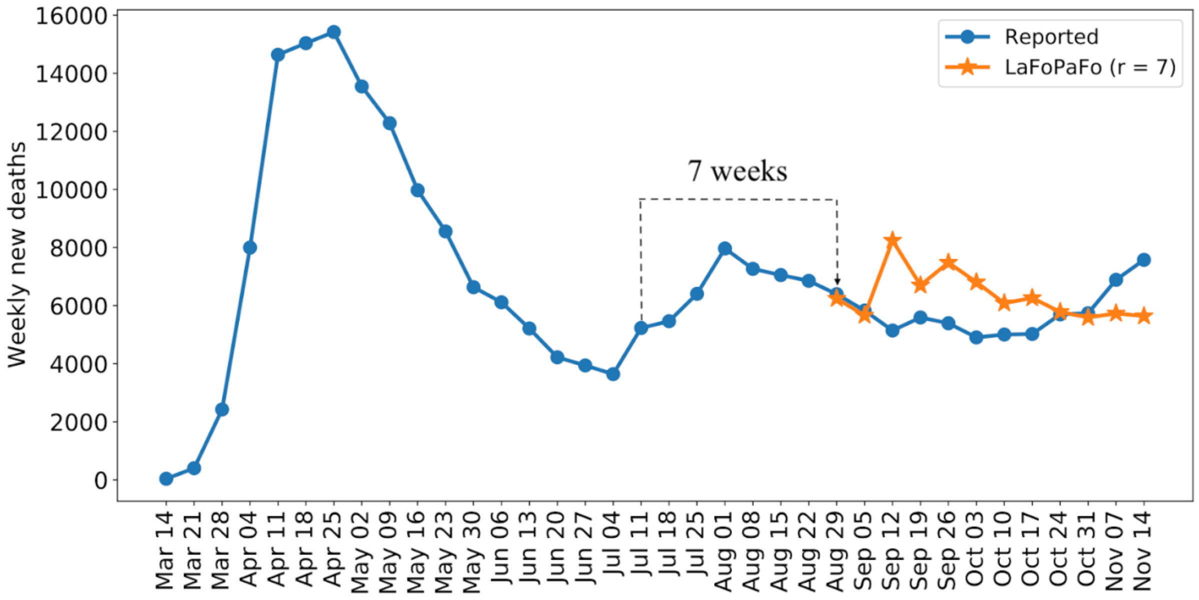

Supplementary Figure 3. LaFoPaFo's future seven-week forecasts of the weekly number of deaths in the US. Each orange point in the graph was predicted seven weeks earlier.

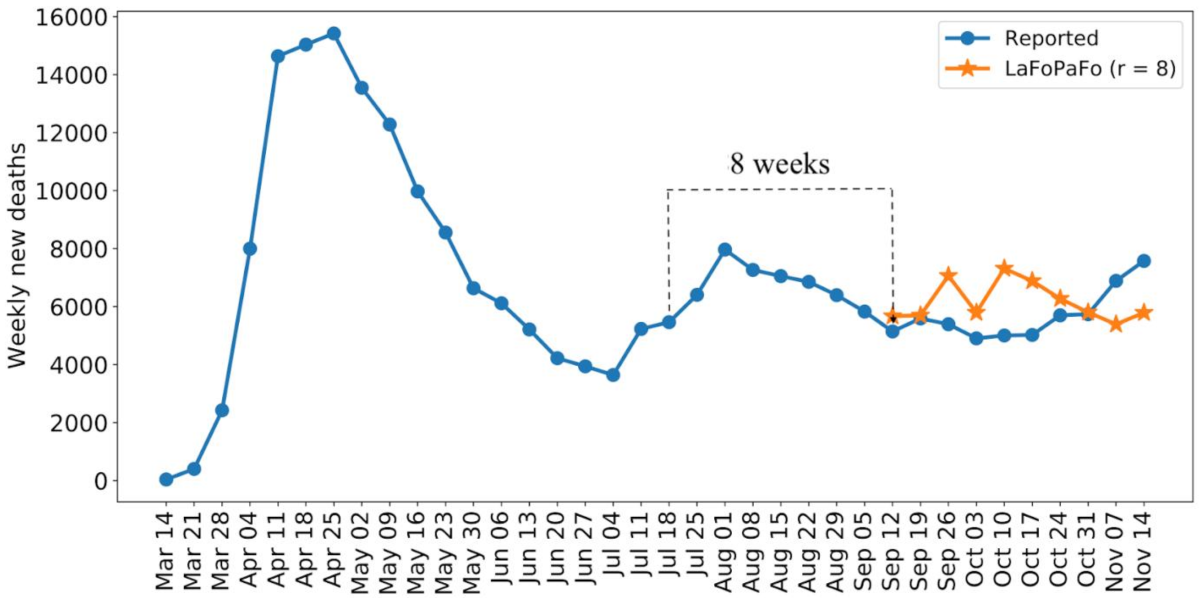

Supplementary Figure 4. LaFoPaFo's future eight-week forecasts of the weekly number of deaths in the US. Each orange point in the graph was predicted eight weeks earlier.

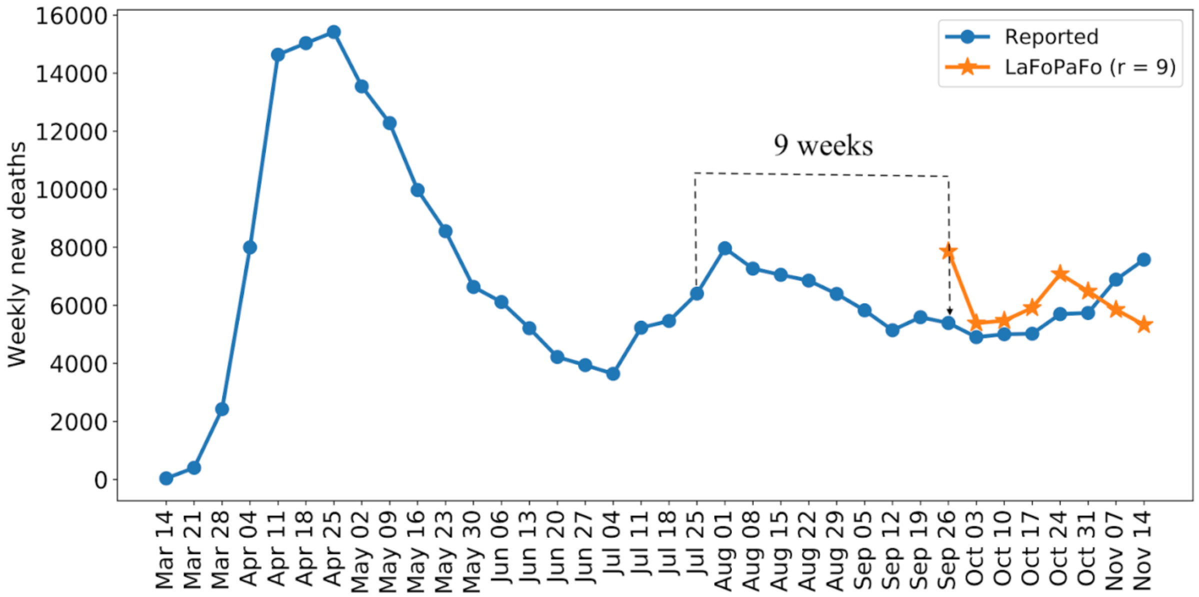

Supplementary Figure 5. LaFoPaFo's future nine-week forecasts of the weekly number of deaths in the US. Each orange point in the graph was predicted nine weeks earlier.

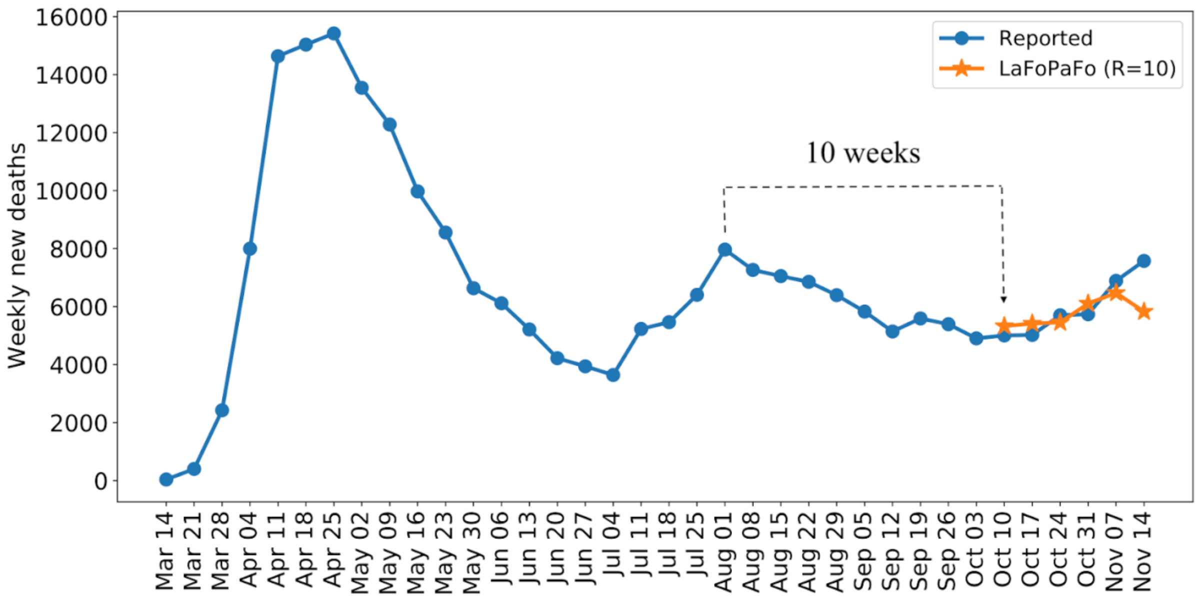

Supplementary Figure 6. LaFoPaFo's future ten-week forecasts of the weekly number of deaths in the US. Each orange point in the graph was predicted ten weeks earlier.

		week t	week t-1	week t-2	week t-3	week t-4
		h = 1	h = 2	h = 3	h = 4	h = 5
Covariate rank	Covariate	May 24--30	May 17--23	May 10--16	May 03--09	Apr 26--May 2
1	daily precipitation	45.73	55.18	27.59	24.98	22.81
2	COVID-19 deaths	948.14	1222.29	1425.00	1754.57	1935.29
3	daily temperature	20.18	17.26	13.30	13.50	14.31
4	daily COVID-19 tests	10303.60	9586.57	8627.52	6904.74	6061.80
5	transit stations	-36.28	-37.57	-39.71	-41.42	-44.57
6	parks	40	23.14	14.42	9.42	1.71
7	workplaces	-37.14	-35.71	-37.57	-39.57	-42.57

8	COVID-19 cases	21039.6	22091.0	22631.3	25266.1	27714.9
9	residences	12.42	12.85	13.85	14.57	15.28
10	grocery stores and pharmacies	-3.57	-3.71	-5.14	-3.57	-10.00
11	retail shops and recreation centers	-23.42	-25.00	-28.85	-30.14	-35.00

Supplementary TABLE 1. Feature set for forecasting #cases, at horizon r =9. LaFoPaFo selects from the covariates at weeks t, t-1, t-2, t-3, and t-4, to forecast the target variable, which here is the number of confirmed cases at week t+9. The value of *t* in the table refers to the week May 24--30, meaning the target variable is the week July 26 -- August 1. The covariates are ranked according to mRMR based on the target variable on the whole train and validation datasets. Here, the target variable for the test instance is on week October 4--10, for the validation is on week September 27 -- October 3, and for the training is from May 17--23 up to the week July 26 -- August 1. LaFoPaFo considers only a limited number of feature combinations for the forecasting task. LaFoPaFo selects the first c* covariates (based on this mRMR ordering) with a history-length h* to construct its final combination of features, which will be its “feature set.” So the feature sets correspond to rectangles in the table starting from the first row and column. Here, LaFoPaFo selected the first seven covariates (c*=7) with three historical time (h*=3), resulting in the blue rectangle. That is, LaFoPaFo produces a model that uses daily precipitation, COVID-19 deaths, daily temperature, daily COVID-19 tests, transit stations, parks, and workplaces on weeks May 10--30 to forecast the number of confirmed cases on week July 26 -- August 1.
